# Assessing the association between ADHD and brain maturation in late childhood and emotion regulation in early adolescence

**DOI:** 10.1038/s41398-025-03411-6

**Published:** 2025-06-02

**Authors:** Kristóf Ágrez, Pál Vakli, Béla Weiss, Zoltán Vidnyánszky, Nóra Bunford

**Affiliations:** 1 Clinical and Developmental Neuropsychology Research Group, HUN-REN Research Centre for Natural Sciences, Budapest, Hungary; 2 Brain Imaging Centre, HUN-REN Research Centre for Natural Sciences, Budapest, Hungary; 3 Machine Perception Research Laboratory, HUN-REN Institute for Computer Science and Control, Budapest, Hungary; 4 Doctoral School of Semmelweis University, Mental Health Sciences Division, Budapest, Hungary

**Keywords:** Neuroscience, Psychology

## Abstract

A delay in brain maturation is a hypothesized pathomechanism of attention-deficit/hyperactivity disorder (ADHD). Differences in emotion regulation are associated with phenotypic and prognostic heterogeneity in ADHD. The development of emotion regulation is driven, in part, by brain maturation. Whether the difference between an individual’s brain age predicted by machine-learning algorithms trained on neuroimaging data and that individual’s chronological age, i.e. brain-predicted age difference (brain-PAD) predicts differences in emotion regulation, and whether ADHD problems add to this prediction is unknown. Using data from the Adolescent Brain Cognitive Development Study, we examined, in 2711 children (*M*_age_ = 120.09 months, *SD* = 7.61; 54.15% female; 61.23% white), whether adjusting for action cancellation (inhibition), age, sex assigned at birth, psychotropic treatment, and pubertal status, brain-PAD in late childhood predicts self-reported emotion regulation in early adolescence (at 3-year follow-up), and whether parent-reported ADHD problems predict self-reported emotion regulation above and beyond brain-PAD. Greater brain-PAD predicted greater expressive suppression (*b* = 0.172, *SE* = 0.051, *p*_FDR_ = 0.004), whereas ADHD problems did not (*b* = 0.041, *SE* = 0.022, *p*_FDR_ = 0.124), model marginal *R*^2^ = 0.020. This pattern of results was replicated across sensitivity tests. Neither brain-PAD, nor ADHD problems predicted cognitive reappraisal, *p*_FDR_s = 0.734. Clinically, consistent with earlier findings linking greater brain-PAD to psychopathology, we observed that greater brain-PAD in childhood—but not ADHD problems—predicted expressive suppression in early adolescence. Expressive suppression is implicated in the etiology, maintenance, and treatment of numerous psychopathologies, highlighting the relevance of brain-PAD in understanding developmental risk mechanisms. Conceptually, these findings further validate brain-PAD as a valuable tool for advancing developmental neuroscience.

Attention-deficit/hyperactivity disorder (ADHD) is a common, functionally impairing, and often persistent disorder [1]. ADHD is mechanistically and phenotypically heterogeneous [2]. Different developmental pathways are hypothesized to lead to observable symptoms and different associated features and negative outcomes are related to such symptoms. A nuanced understanding of the link between specific pathophysiological mechanisms and specific associated features is necessary for effective early identification and personalized prevention.

A delay in brain maturation is one hypothesized pathomechanism of ADHD. Although the progression of brain maturation in children with ADHD parallels the progression of brain maturation in children without ADHD, in children with ADHD, there is an approximately three-year delay in the rate of brain maturation [3].

Defined and measured across studies in various ways, emotion regulation is an associated feature of ADHD. Across development, ADHD is associated with difficulties with emotion regulation [2, 4] and there is evidence that genetic risk for ADHD is associated with differences in dimensions of temperament related to emotion regulation [5]. Emotion regulation is a key feature of ADHD, because it is prognostically relevant. In ADHD, whereas difficulties with emotion regulation exacerbate and explain the development of comorbidities including depression; functional impairment including in interpersonal relationships with parents and peers; and negative outcomes including alcohol misuse [2, 4], adaptive emotion regulation is one of a few identified factors of resilience [6].

Development of emotion regulation skills is driven by brain maturation and by experience. The development of emotion regulation is biologically governed by maturation of frontal-striatal circuitry such that areas implicated in emotional control (e.g., prefrontal cortex) undergo protracted development across early childhood and adolescence and exert increasing control over areas implicated in emotional reactivity (e.g., amygdala) [7, 8]. The development of emotion regulation is experientially shaped by interactions with caregivers and peers [9, 10].

A reliable and valid [11] measure of brain maturation is the difference between brain age and chronological age (brain-PAD), defined as the difference between an individual’s brain age predicted by machine learning algorithms trained on neuroimaging data and that individual’s chronological age. Whether brain-PAD predicts differences in emotion regulation and whether ADHD problems add to this prediction is unknown.

## Current study

Our aim in the current study was to determine whether differences in brain maturation in late childhood account for differences in later emotion regulation in early adolescence and whether ADHD problems add to this prediction. Specifically, we examined, in a large sample of 9–10-year-old children, whether brain-PAD measured at baseline is associated with self-rated cognitive reappraisal and expressive suppression measured at 3-year follow-up, and whether parent-rated ADHD problems account for additional variance in these indices of emotion regulation, adjusting for covariates. We used a publicly available, state-of-the-art deep learning model, the regression variant of the Simple Fully Convolutional Network [12] for brain age estimation. This 3-dimensional convolutional neural network was developed using a large and diverse sample of T1-weighted magnetic resonance imaging (MRI) scans (*N* = 53542) that underwent minimal preprocessing—including skull-stripping and spatial normalization—and showed excellent generalization performance on novel data from unseen scanners.

Specifically, brain age is inherently related to chronological age [13] and is correlated with pubertal status [11]. Accordingly, to examine whether any observed relations between brain age and emotion regulation are not better explained by an association between chronological age and emotion regulation or pubertal status and emotion regulation, we account for chronological age and pubertal status.

Furthermore, findings indicate age-related [1, 14], ethnic, racial [15, 16], and sex-related [17, 18] differences in ADHD manifestation, prevalence, and severity and also in emotion regulation. ADHD is often comorbid with a range of psychological problems and symptoms, including externalizing and internalizing problems [1]. Emotion regulation, by virtue of being a transdiagnostic construct, is also a correlate or mechanism of such psychopathologies [1, 4]. Most pharmacotherapeutic agents indicated for mental disorders, including ADHD, affect emotion regulation [19, 20]. Both ADHD and emotion regulation are associated with behavioral inhibition [1, 4], crystallized and fluid intelligence [21, 22] and with pubertal maturation [23]. In sensitivity analyses, we account for each of these key characteristics relevant to the relation of brain-PAD and of ADHD problems with emotion regulation in a stepwise manner.

## Methods

Participants of the current study were participants from the Adolescent Brain Cognitive Development (ABCD) Study, a nationwide, 21-site research carried out in the United States. ABCD protocols, including recruitment strategies, are extensively documented in the literature [24]. Tabulated data were acquired from Annual Release 5.1 [25].

### Ethics approval and consent to participate

All methods were performed in accordance with the relevant guidelines and regulations [26]. The ABCD Study was approved by a central Institutional Review Board at the University of California, San Diego and each site also obtained approval from their respective Institutional Review Boards. Parents or guardians provided written informed consent, and children provided assent.

### Sample

Participants were excluded in case of parent-reported: prenatal illicit substance exposure, including marijuana, crack/cocaine, heroin/morphine, oxycontin, amphetamines or methamphetamine, benzodiazepines, cathinones, GHB, hallucinogens, inhalants, ketamine, MDMA, opioids, barbiturates, or other substances, or if this information was not available (“Don’t know”) on the Developmental History Questionnaire [27] at baseline or the 4-year follow-up (for a subset of participants with data available) (excluding *n* = 637); head injury resulting in: loss of consciousness lasting for more than 30 min, and/or in difficulties remembering lasting for longer than a day, and/or in difficulties getting a brain scan on the ABCD screener interview at baseline (excluding *n* = 255); head or neck injury that required hospitalization or treatment in an emergency room on the short version of the modified Ohio State Traumatic Brain Injury Screen [28] at baseline (excluding *n* = 1179); or if parent- or youth-reported diagnostic criteria were met for Unspecified Schizophrenia Spectrum and Other Psychotic Disorder on the Kiddie Schedule for Affective Disorders and Schizophrenia at baseline, 1-year or 2-year follow-up (excluding *n* = 162). For demographic and descriptive statistics of the sample, see Table 1.

**Table 1:** Key characteristics of the sample at baseline (*N* = 2711).

	*M* (*SD*) or *n* (%)
Age (in months)	120.089 (7.609)
Sex assigned at birth	
Male	1243 (45.85%)
Female	1468 (54.15%)
Race/Ethnicity	
Asian	52 (1.92%)
Black	228 (8.41%)
Hispanic	474 (17.48%)
White	1660 (61.23%)
Other	297 (10.96%)
Pubertal stage (based on PDS)	
Prepuberty	1408 (51.94%)
Early puberty	663 (24.46%)
Mid puberty	611 (22.54%)
Late puberty	29 (1.07%)
Cognition	
Crystallized Composite (Age-Corrected Standard Score)	109.874 (18.306)
Fluid Composite (Age-Corrected Standard Score)	99.867 (16.134)
Present ADHD diagnosis^a^	
Yes	123 (4.54%)
No	2588 (95.45%)
Pharmacotherapy	
ADHD	166 (6.12%)
Non-ADHD (psychotropic)	56 (2.07%)
Total combined family income^b^	
Less than $5,000	51 (1.88%)
$5,000 through $11,999	41 (1.51%)
$12,000 through $15,999	38 (1.40%)
$16,000 through $24,999	88 (3.25%)
$25,000 through $34,999	106 (3.91%)
$35,000 through $49,999	216 (7.97%)
$50,000 through $74,999	316 (11.66%)
$75,000 through $99,999	428 (15.79%)
$100,000 through $199,999	887 (32.35%)
$200,000 and greater	386 (14.24%)
Don’t know	76 (2.80%)
Prefer not to answer	88 (3.25%)
Highest level of education (primary caregiver)	
Below high school	108 (3.98%)
High school graduate, GED or equivalent diploma	202 (7.45%)
Bachelor’s or Associate degree	1590 (58.65%)
Master’s degree	604 (22.28%)
Doctoral or Professional School degree	206 (7.60%)
Prefer not to answer	1 (0.04%)

*ADHD* attention-deficit/hyperactivity disorder, *PDS* pubertal development scale.

^a^Based on parent-report on the self-administered, computerized version of the Kiddie Schedule for Affective Disorders and Schizophrenia.

^b^Percentages may not add up to 100% due to rounding.

### MRI acquisition and preprocessing

Minimally processed MRI data were acquired from Release 5.0, published in June, 2023. Image acquisition and centralized minimal preprocessing of neuroimaging data is described in detail elsewhere [29]. T1-weighted images were processed if recommended for inclusion, were present in the fmriresults01 table (excluding *n* = 978), and if no abnormal findings requiring clinical referral were reported (excluding *n* = 375). Participants with an average framewise displacement above 0.15 mm during resting-state (used as a proxy for tendency to move during the T1-weighted scan) were excluded [30] (excluding *n* = 4863), leaving a sample of *n* = 3418 for MRI processing.

Minimal preprocessing was applied to the T1-weighted images using the preprocessing pipeline developed by Leonardsen et al. [12]. In particular, images were skull-stripped using the FreeSurfer 5.3 auto-recon pipeline [31], reoriented to standard FSL [32] orientation using fslreorient2std, and linearly registered to the MNI152 space using FLIRT [33] using linear interpolation and the default 1 mm FSL 6.0 template. Images were cropped by removing voxels outside the [6:172, 2:213, 0:159] range, and voxel intensity values were normalized to the range [0, 1]. Visual inspection revealed that preprocessing was performed reasonably well for the vast majority of the images, with the exception of 21 scans where skull-stripping was only partially completed. These images were excluded from analysis. Additionally, participants without parent-reported ADHD diagnostic information on the Kiddie Schedule for Affective Disorders and Schizophrenia were also excluded (*n* = 65).

The Euler number [34], derived from FreeSurfer 7.1.1, was used for in-house quantitative image quality assessment [35]. Values were normalized [36], and participants with normalized Euler numbers ≤ first quartile – 1.5 * interquartile range were flagged as outliers and excluded (*n* = 15), with *n* = 3317 T1-weighted images available for brain age estimation.

### Measures

#### Brain age estimation

The Simple Fully Convolutional Network model [12] used for brain age prediction consists of 5 repeated convolutional blocks of three-dimensional convolution (with a filter size of 3 × 3 × 3), batch normalization [37], rectified linear unit activation [38], and max pooling (with a pooling size of 2 × 2 × 2). The last convolutional block is followed by pointwise convolution, batch normalization, rectified linear unit activation, and global average pooling, and then a single output unit with linear activation. The model was developed using a large and diverse cohort of 21 publicly available datasets (*N* = 53542; female *n* = 27715; age range = 3–95 years). On an external dataset (*N* = 2554; female *n* = 1371; age range = 13–96 years), the model achieved a mean absolute error of 3.90 years [12]. The brain-predicted age difference (brain-PAD), calculated as the difference between the predicted and chronological age, was used in statistical analyses. In the current sample (*n* = 3317), predicted brain ages had a mean absolute error of 0.829 years, a root-mean square error of 1.129, and brain-PAD values ranged from −2.022 to 7.082, *M* = 0.256 (*SD* = 1.100).

#### ADHD problems

The Attention Deficit/ Hyperactivity (ADH) Problems subscale raw score of the Child Behavior Checklist (CBCL) [39] was used as an index of ADHD-related problems and symptomatology. The CBCL is a 113-item parent-rated measure of behavioral and emotional problems in children and adolescents. Items are rated on a three-point scale ranging from 0 (absent) to 2 (occurs often). Higher scores indicate more frequent problems. DSM-oriented and syndrome scales can be calculated, including the ADH Problems DSM-oriented scale, which is comprised of seven items assessing behaviors commonly associated with ADHD, including hyperactivity, impulsivity, and inattention. Evidence indicates the ADH Problems subscale exhibits acceptable reliability, concurrent and discriminant validity, diagnostic efficiency, and acceptable sensitivity in comparison to a clinical interview [40]. The ADH Problems subscale exhibited acceptable internal consistency, ω = 0.839 (α = 0.833).

#### Emotion regulation

The cognitive reappraisal and the expressive suppression subscales of the Emotion Regulation Questionnaire for Children and Adolescents [41] were used to assess self-reported emotion regulation. Items are rated on a five-point scale, ranging from 1 (strongly disagree) to 5 (strongly agree). The cognitive reappraisal subscale (three items) is a measure of adaptive emotion regulation; items assess the respondent’s ability to change their thinking about an emotion-eliciting situation to alter its emotional impact. The expressive suppression subscale (three items) is a measure of maladaptive emotion regulation; items assess the respondent’s tendency to inhibit emotion expression. Higher scores indicate more reappraisal and suppression. Evidence indicates the Emotion Regulation Questionnaire subscales exhibit acceptable reliability and validity. Both subscales exhibited acceptable internal consistency, ω_reappraisal_ = 0.737 (α = 0.717), ω_suppression_ = 0.778 (α = 0.775).

#### Demographics

Child chronological age, ethnicity/race, were assessed using the PhenX Toolkit [42], sex assigned at birth using the Pubertal Development Scale (PDS) [43].

#### ADHD diagnostic status

ADHD diagnoses were established using the computerized version of a semi-structured diagnostic interview, the Kiddie Schedule for Affective Disorders and Schizophrenia (KSADS) for DSM-5 (KSADS-5) [44]. Presence of select ADHD symptoms is assessed in a screening interview and if a screening symptom is positive, duration, impairment and severity are assessed in supplemental probes. Data were collected via the self-administered computerized version from the caregiver/parent [27]. Evidence indicates the KSADS exhibits acceptable reliability and validity [44] and in case of the KSADS-5 computerized version, percent agreement between the clinician-administered paper-and-pencil version and the self-administered computerized version for diagnostic categories ranges from 88–96%, and kappas range from good to excellent [45].

#### Behavioral and emotional problems/ Psychopathology symptoms

The Total Problems raw score without the ADH Problems raw score of the CBCL was used as an index of psychological problems and symptoms. The Total Problems score is derived from all CBCL items and, as such, is a measure of externalizing problems, internalizing problems, and other behavioral issues. Specifically, the Total Problems score combines scores from syndrome scales including Aggressive Behavior, Rule-Breaking Behavior, Anxious/Depressed, Withdrawn/Depressed, Somatic Complaints as well as problem scales including Attention Problems, Sleep Problems, Social Problems, and Thought Problems. Higher scores indicate more frequent problems. The Total Problems (without ADH Problems items) scale exhibited acceptable internal consistency, ω = 0.931 (α = 0.930).

#### Action cancellation (inhibition)

The Stop Signal Reaction Time (SSRT), calculated using mean estimation, was used as an index of cognitive control from the Stop Signal Task [46].

#### Psychotropic medications

Current medications were assessed by a Medication Inventory from the PhenX Toolkit. Each medication was categorized based on being indicated for the treatment of ADHD or for another mental disorder. Medications were primarily categorized using MedlinePlus [47], secondary resources included the NHS [48] or the official websites of manufacturers.

#### Crystallized and fluid intelligence

The NIH Toolbox® cognition measures were used to assess cognitive performance. The Picture Vocabulary Task and the Oral Reading Recognition Task to measure crystallized intelligence and the Dimensional Change Card Sort Test, Flanker Inhibitory Control and Attention Test, List Sorting Working Memory Test, Pattern Comparison Processing Speed Test, and Picture Sequence Memory Test to measure fluid intelligence. Age-corrected standard scores were used in analyses.

#### Pubertal status

Classification based on the PDS was used to assess parent-reported pubertal status. The PDS is a five-item self-, and parent-report measure of physical maturity in children and adolescents. In boys, items assess body hair, facial hair, changes in skin, changes in voice, and growth spurt. In girls, items assess body hair, changes in skin, breast development, growth spurt, and menarche. Items are rated on a four-point scale, ranging from 1 (no development) to 4 (completed development). Based on their responses, respondents can be classified as being in 1 - prepuberty, 2 - early puberty, 3 - mid puberty, 4 - late puberty, 5 - post puberty. Evidence indicates the PDS exhibits acceptable reliability and validity, including when compared to physical examination [43, 49]. In the current sample, the PDS exhibited unacceptable internal consistency, ω = 0.472 (α = 0.396) for participants assigned female at birth, ω = 0.388 (α = 0.317) for participants assigned male at birth.

### Analytic plan / Statistical analyses

Statistical analyses were carried out in R (v4.4.1) within RStudio (v2024.09.0 + 375). McDonalds’s omega (ω) is reported as the primary internal consistency metric, although Cronbach’s alpha (α) is also reported to aid cross-study comparison. These metrics were calculated with the psych (v2.4.6.26) [50] package.

Linear mixed effects models were fit to account for the non-independent nature of the data using lme4 (v1.1-35.5) [51], including in each model a nested random intercept, with family identifiers (variable name: rel_family_id) nested within hashed identifiers of magnetic resonance imaging scanners (variable name: mri_info_deviceserialnumber). Quantile-quantile plots indicated departures from normality which were addressed with fitting robust linear mixed effects models as implemented in robustlmm (v3.3-1) [52]. Robustlmm capitalizes on the Design Adaptive Scale approach [53] and robust scoring equations [54]. Tuning parameters were set to the recommended values (setting “RSEn”). Significance levels for robust models were obtained with sjPlot (v2.8.16) [55], and *p*-values of interest, i.e., *p*-values of brain-PAD values and CBCL ADH Problems raw scores, were False Discovery Rate (FDR) [56] adjusted. For models with significant variables of interest, marginal *R*^2^ is reported to quantify model explanatory power. For multicategorical covariates, reference categories were white (race/ethnicity), and prepuberty (pubertal status).

As only a single person was reported to be post pubertal, they were excluded from analysis.

To assess the robustness of the results, sensitivity analysis was conducted with seven alternative sets of independent variables, see Supplementary Tables S2–S15. Alternative independent variable sets included (new variables compared to the preceding model are highlighted with bolding):

1. Brain-PAD, age, normalized Euler number (Tables S2 and S9);
2. Brain-PAD, age, **CBCL ADH Problems (raw scores)**, normalized Euler number, **pharmacotherapy: ADHD,** **pharmacotherapy: non-ADHD (psychotropic),** **sex assigned at birth** (Tables S3 and S10);
3. Brain-PAD, age, CBCL ADH Problems (raw scores), normalized Euler number, pharmacotherapy: ADHD, pharmacotherapy: non-ADHD (psychotropic), sex assigned at birth, **SSRT** (Tables S4 and S11);
4. Brain-PAD, age, CBCL ADH Problems (raw scores), **CBCL Total (excluding ADH) Problems**, normalized Euler number, pharmacotherapy: ADHD, pharmacotherapy: non-ADHD (psychotropic), sex assigned at birth, SSRT (Tables S5 and S12);
5. Brain-PAD, age, CBCL ADH Problems (raw scores), CBCL Total (excluding ADH) Problems, normalized Euler number, pharmacotherapy: ADHD, pharmacotherapy: non-ADHD (psychotropic), **pubertal status**, sex assigned at birth, SSRT (Tables S6 and S13);
6. Brain-PAD, age, CBCL ADH Problems (raw scores), CBCL Total (excluding ADH) Problems, **crystallized intelligence (age-corrected standard score),** **fluid intelligence (age-corrected standard score)**, normalized Euler number, pharmacotherapy: ADHD, pharmacotherapy: non-ADHD (psychotropic), pubertal status, sex assigned at birth, SSRT (Tables S7 and S14);
7. Brain-PAD, age, CBCL ADH Problems (raw scores), CBCL Total (excluding ADH) Problems, crystallized intelligence (age-corrected standard score), fluid intelligence (age-corrected standard score), normalized Euler number, pharmacotherapy: ADHD, pharmacotherapy: non-ADHD (psychotropic), pubertal status, **race/ethnicity**, sex assigned at birth, SSRT (Tables S8 and S15).

After excluding participants with missing data, the final, analytic sample consisted of *n* = 2711 participants.

Considering the relative complexity of mixed effects models, a simulation-based approach is recommended for statistical power analysis [57]. Simulations are informed by prior studies and/or a priori knowledge about the model in question. As simulations are only as informative as their data (or priors) are representative the model for which the power analysis is conducted, in the absence of prior studies to inform our investigation, we did not conduct a power analysis.

## Results

### Emotion regulation strategies

Neither baseline brain-PAD (*b* = −0.015, *SE* = 0.043, *p*_FDR_ = 0.734) nor baseline ADH Problems (*b* = 0.009, *SE* = 0.019, *p*_FDR_ = 0.734) were associated with cognitive reappraisal at 3-year follow-up. Of covariates, only non-ADHD pharmacotherapy (psychotropic) (*b* = −0.674, *SE* = 0.321, *p*_unadj._=0.036) was negatively associated with cognitive reappraisal at 3-year follow-up (Table 2).

**Fig. 1:**
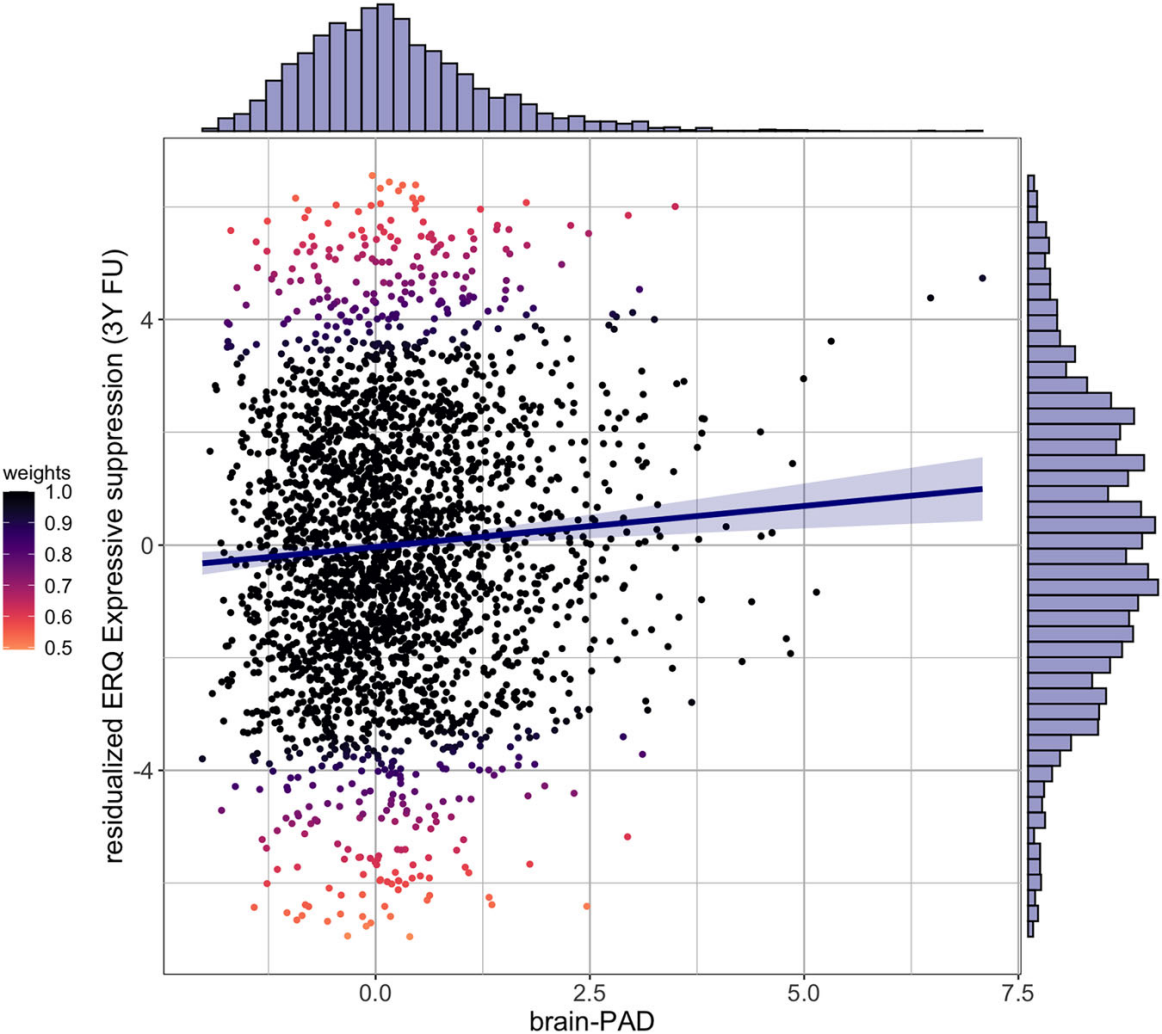
Greater brain-PAD in childhood predicts greater expressive suppression in early adolescence. 3Y FU 3-year follow-up; brain-PAD brain-predicted age difference, ERQ emotion regulation questionnaire for Children and Adolescents. Residualized ERQ Expressive suppression scores are created by regressing all independent variables but brain-PAD (fixed effects: CBCL ADHD Problems, Pharmacotherapy: ADHD, Pharmacotherapy: non-ADHD, age, pubertal development scale scores, sex assigned at birth, and normalized Euler number; random intercept: family identifiers nested within scanner identifiers) onto ERQ expressive suppression values.

Baseline brain-PAD was positively associated with expressive suppression at 3-year follow-up (*b* = 0.172, *SE* = 0.051, *p*_FDR_ = 0.004), but ADH Problems were not (*b* = 0.041, *SE* = 0.022, *p*_FDR_ = 0.124). Of covariates, chronological age (*b* = 0.026, *SE* = 0.007, *p*_unadj_. < 0.001), being in mid (*b* = 0.466, *SE* = 0.148, *p*_unadj._ = 0.002) or late (*b* = 1.238, *SE* = 0.504, *p*_unadj_. = 0.014) puberty as compared to prepuberty were positively associated with expressive suppression, whereas being on non-ADHD pharmacotherapy (psychotropic) (*b* = −0.818, *SE* = 0.372, *p*_unadj._ = 0.028) was negatively associated with expressive suppression at 3-year follow-up (Table 2 and Fig. 1). Fixed effects explained 2% variance of expressive suppression. Fixed effects of the same model without brain-PAD explained 1.5% variance of expressive suppression (Table S1).

**Table 2:** Coefficients for main robust regression models.

	ERQ Cognitive reappraisal at 3-year follow-up					
	*b*	*SE*	95% CI		*t*	*p*
			*LL*	*UL*		
(Intercept)	9.879	0.868	8.178	11.581	11.384	<0.001
Brain-PAD	−0.015	0.043	−0.099	0.070	−0.339	0.734
CBCL ADH Problems (raw scores)	0.009	0.019	−0.028	0.046	0.490	0.624
Pharmacotherapy: ADHD	−0.325	0.207	−0.731	0.081	−1.568	0.117
Pharmacotherapy: non-ADHD	−0.674	0.321	−1.302	−0.045	−2.102	0.036
Age (in months)	0.003	0.006	−0.009	0.015	0.499	0.618
PDS: early puberty	0.028	0.110	−0.188	0.244	0.255	0.799
PDS: mid puberty	0.228	0.126	−0.018	0.475	1.814	0.070
PDS: late puberty	0.350	0.433	−0.499	1.198	0.809	0.419
Sex assigned at birth: female	−0.008	0.100	−0.204	0.189	−0.077	0.939
Normalized Euler number	0.021	0.100	−0.175	0.218	0.214	0.831
	Marginal *R*^2^ = 0.006					

	ERQ Expressive suppression at 3-year follow-up					
	*b*	*SE*	95% CI		*t*	*p*
			*LL*	*UL*		
(Intercept)	6.047	1.023	4.040	8.054	5.908	<0.001
Brain-PAD	0.172	0.051	0.071	0.272	3.342	0.001
CBCL ADH Problems (raw scores)	0.041	0.022	−0.002	0.084	1.870	0.062
Pharmacotherapy: ADHD	0.131	0.241	−0.342	0.603	0.541	0.588
Pharmacotherapy: non-ADHD	−0.818	0.372	−1.546	−0.089	−2.201	0.028
Age (in months)	0.026	0.007	0.012	0.040	3.536	<0.001
PDS: early puberty	0.181	0.128	−0.070	0.433	1.413	0.158
PDS: mid puberty	0.466	0.148	0.175	0.757	3.139	0.002
PDS: late puberty	1.238	0.504	0.249	2.227	2.455	0.014
Sex assigned at birth: female	−0.094	0.116	−0.322	0.134	−0.808	0.419
Normalized Euler number	0.020	0.118	−0.212	0.252	0.170	0.865
	Marginal *R*^2^ = 0.020					

Non-ADHD pharmacotherapy is limited to psychotropic therapeutic agents. *p*-values are unadjusted for multiple comparisons.

*ADHD* attention-deficit/hyperactivity disorder, *b* unstandardized regression coefficient, *Brain-PAD* brain-predicted age difference, *CBCL* child behavior checklist, *CI* confidence interval, *ERQ* emotion regulation questionnaire for children and adolescents, *LL* lower limit, *PDS* pubertal development scale, *SE* standard error, *UL* upper limit.

In sensitivity analyses, brain-PAD was not associated with cognitive reappraisal (Tables S2–S8), but it was positively associated with expressive suppression (Tables S9–S15).

## Discussion

Our aim in this research was to examine whether brain-PAD in late childhood prospectively predicts self-reported cognitive reappraisal and expressive suppression in early adolescence and whether parent-rated ADHD problems add to this prediction. Across main and sensitivity analyses, brain-PAD predicted self-reported expressive suppression, such that greater brain-PAD was associated with greater expressive suppression.

Brain age estimation was originally proposed as a tool to assess cognitive deficits and diseases related to ageing and, as such, it has most often been examined in older populations [58–60]. In older populations, the interpretation of brain age estimates is relatively straightforward as a brain that is “older” than what is expected for a given chronological age is reflective of atypical degeneration. Indeed, in adults and older adults, a greater brain-PAD has been linked to Alzheimer’s disease, cognitive aging and decline, and with anxiety, bipolar, and major depressive disorders and schizophrenia.

In youth, findings indicate brain-PAD is associated with known metrics of puberty and, across time, tracks with those metrics [11]. Yet, available findings on the relation of brain age with cognition and psychopathology are mixed. Some indicate that a greater brain-PAD is associated with faster processing speed [61], whereas others show that a lower brain-PAD is associated with better cognitive performance [62] and yet others show no relation [63–66]. Regarding psychopathology, a greater brain-PAD is associated with depression, obsessive-compulsive symptoms, and psychosis [67–69]. Here, we found that a greater brain-PAD is associated with greater expressive suppression, an emotion regulation strategy involving the inhibition of overt emotional expression once an emotional response has begun. This finding is consistent with the general hypothesis that atypical developmental trajectories contribute to the development of, or confer risk for, mental disorders [70]. The significance of this result is both conceptual and methodological. First, this finding suggests that differences in brain maturation explain differences in maladaptive emotion regulation, a characteristic that plays a role in the etiology, maintenance, and prognosis of, and is an intervention target for, many disorders beyond ADHD, including anxiety, depressive, borderline personality, posttraumatic stress, and substance use disorders [71, 72]. Second, this finding further validates brain age estimates as a tool to advance affective and developmental neuroscience.

Across models, the association between brain-PAD and emotion regulation was apparent for expressive suppression but not for cognitive reappraisal, indicating that the association between brain maturation and emotion regulation may be specific to precocious brain maturation and maladaptive emotion regulation.

There is reason to believe that there are aspects or correlates of ADHD that account for variance in emotion regulation beyond brain maturation. Specifically, the development of emotion regulation is driven and supported by a complex interaction between biological, environmental, and social factors. Whereas ADHD can be linked to many of these biological, environmental, and social factors, brain maturation is only one of the relevant biological influences.

Regarding biological factors, differences in genetics and early-evident temperamental traits are associated with differences in emotion regulation [2]. The genetic and temperamental correlates of ADHD overlap with those of emotion regulation [5]. Regarding environmental and social factors, differences in early experiences and stressors (including trauma), attachment, parenting practices, peer relationships, are also associated with differences in emotion regulation [73]. Childhood maltreatment [74] and less adaptive and constructive parenting [75] are more common in the families of children with ADHD, and impairments in peer relationships [4] are associated with ADHD. As early life stress may accelerate neural development (leading to short-term benefits but long-term vulnerabilities) [76], early stress is relevant to both brain development and ADHD. Conversely, the impairments in peer relationships that impact emotion regulation are arguably less directly relevant to brain maturation than to ADHD. For example, peers can model emotion regulation, provide feedback that can enhance emotion regulation, and positive peer interactions can promote adaptive emotional responses [10]. As children with ADHD are more likely to affiliate with deviant peers and with younger peers, to be rejected by peers, and are less likely to accurately interpret and recognize social cues [77], ADHD-associated impairments in peer relationships may directly hinder development of age-appropriate and adaptive emotion regulation skills. With these considerations taken together, it stands to reason that there are aspects of ADHD that will account for additional variance in emotion regulation beyond brain maturation.

Yet, across main and sensitivity analyses, - despite no common method variance between measures of brain age and expressive suppression and shared method variance between the measure of ADHD problems and expressive suppression –, parent-reported ADHD problems did not add to the prediction by brain-PAD of self-reported expressive suppression. A limited range in ADHD problems may explain a weak (nonsignificant) association between ADHD problems and expressive suppression. However, in the model with brain-PAD, chronological age, ADHD problems, ADHD and non-ADHD pharmacotherapy (psychotropic), and sex assigned at birth, ADHD problems ranged from 0–14 (with 14 being the highest possible on the scale), and 1097 participants had a score of zero, 1170 had scores between one and four, 411 participants had scores between five and ten, and 33 participants had scores between 11 and 14, indicating scores were variable. It thus appears that, at least based on the findings obtained in the current, mostly general population sample, ADHD does not add to the explanation of expressive suppression in children beyond what is explained by precocious brain maturation.

Because the association between variables can vary as a function of the covariates that are included in models [78], we conducted considerable sensitivity testing with covariates entered separately. Findings for brain-PAD and ADHD problems were replicated with age, ethnicity/ race, sex assigned at birth, pubertal status, cognitive functioning, inhibition, pharmacotherapy, and behavioral and emotional symptoms covaried in different groupings, indicating that the herein observed pattern of results are robust.

Across analyses, in addition to brain-PAD, chronological age also consistently predicted expressive suppression. As noted, the development of emotion regulation skills is driven, biologically, by brain maturation and experientially, by experiences. Chronological age in this context can be conceptualized as a proxy of experience. The only other variable – albeit not examined across all models – that consistently predicted expressive suppression was non-ADHD pharmacotherapy (psychotropic). This finding is not unexpected as the majority of medications used to treat mental disorders, e.g. antidepressants [19] or antipsychotics [79], affect emotion regulation circuitry [79] and improve behavioral emotion regulation, including expressive suppression [19]. In the presence of brain-PAD and chronological age (and adjusted for all other variables), whether pubertal status predicted expressive suppression depended on the other covariates included in the model and behavioral and emotional symptoms, cognitive functioning, and sex assigned at birth did not predict expressive suppression indicating that the effect of these covariates on the outcome is either confounded, less robust or weak. In line with the available literature, relative to White children, being Black and being Hispanic were associated with greater expressive suppression in early adolescence [16].

Brain-PAD and behavioral response inhibition were differentially associated with emotion regulation. Whereas brain-PAD predicted expressive suppression (but inhibition did not), inhibition predicted cognitive reappraisal (but brain-PAD did not). Behavioral inhibition is a higher-order cognitive function closely tied to cognitive control processes, primarily governed by the dorsolateral prefrontal cortex. This function places substantial demands on cognitive resources. In contrast, the expressive suppression subscale of the Emotion Regulation Questionnaire for Children and Adolescents reflects relatively simpler processes that require less cognitive effort (e.g., “*I control my emotions by not expressing them*”). Expressive suppression has been associated with increased gray matter volume in regions such as the dorsal anterior cingulate cortex and dorsomedial prefrontal cortex [80]. It is also consistently linked to activation in the frontoparietal network, particularly the inferior parietal cortex, and ventrolateral prefrontal cortex [81]. On the other hand, items on the cognitive reappraisal subscale reflect more complex, higher-order processes that are cognitively demanding (e.g., “*I control my emotions by changing the way I think about the situation I’m in*”). These processes are known to engage the dorsolateral prefrontal cortex more extensively [81].

### Directions for future research

Although in the current sample, ADHD did not add to the explanation of expressive suppression beyond precocious brain maturation, ADHD may add to such explanation in e.g. adolescent, adult, or clinical samples.

As prior findings in the ABCD cohort indicate differences between children with high relative to low-noise MRI data in demographic, psychological, and psychiatric traits [30], better data quality likely also resulted in a clinically less representative sample in the current study.

Although the brain age prediction model was developed using a diverse and large cohort of 53,542 MRI scans from healthy individuals aged between 3 and 95 years, the majority of the participants in that cohort were over 45 years old. Future investigations should examine the relation between brain maturation and emotion regulation using models that are more representative of participants under 20 years of age, as new datasets and foundational brain age prediction models become available.

Here, we applied a measure of emotion regulation that represented one domain of the broad and multifaceted construct and one measurement modality. Future research may assess other aspects and measurement modalities. It will also be relevant to examine whether differences in brain age predict changes in emotion regulation over time. Sex differences, especially at the level of specific brain regions, also warrant further investigation.

We also note strengths of our study. Analyses were conducted with a large sample. We applied well-validated measures of emotion regulation and ADHD problems. Comprehensive sensitivity testing ensured that our findings are not dependent on the way in which covariates were selected.

## Supplementary information

Supplemental Material

## Data Availability

Data from the ABCD Study is available to qualified researchers. For more details, please visit https://abcdstudy.org/scientists/data-sharing/.

## Code availability

The R code used for statistical analysis is available from the corresponding author on reasonable request.
