# Supplementary material for: Assessing the association between ADHD and brain maturation in late childhood and emotion regulation in early adolescence
Source: Transl Psychiatry. 2025 Jun 2;15:185. doi: 10.1038/s41398-025-03411-6 (PMC12130550; doi:10.1038/s41398-025-03411-6)
Supplement: Supplementary file 1 — Supplemental Material [file 41398_2025_3411_MOESM1_ESM.docx]

SUPPLEMENTARY INFORMATION TO:

Assessing the association between ADHD and brain maturation in late childhood and emotion regulation in early adolescence

**Supplementary Table Legends**

**Table S1.** *Regression coefficients of the main expressive suppression model without brain-PAD.*

*Notes*. *b*=unstandardized regression coefficient; Brain-PAD=brain-predicted age difference; CI=confidence interval; *LL*=lower limit; PDS=Pubertal Development Scale; *p*-values are unadjusted for multiple comparisons; *SE*=standard error; *UL*=upper limit. Non-ADHD pharmacotherapy is limited to psychotropic therapeutic agents.

**Table S2.** *Regression coefficients of the cognitive reappraisal model with brain-PAD and chronological age.*

*Notes*. *b*=unstandardized regression coefficient; Brain-PAD=brain-predicted age difference; CI=confidence interval; *LL*=lower limit; *p*-values are unadjusted for multiple comparisons; *SE*=standard error; *UL*=upper limit.

**Table S3.** *Regression coefficients of the cognitive reappraisal model with brain-PAD, and chronological age, pharmacotherapy, and sex assigned at birth.*

*Notes*. ADHD=Attention-deficit/hyperactivity disorder; *b*=unstandardized regression coefficient; Brain-PAD=brain-predicted age difference; CBCL=Child Behavior Checklist; CI=confidence interval; *LL*=lower limit; *p*-values are unadjusted for multiple comparisons; *SE*=standard error; *UL*=upper limit. Non-ADHD pharmacotherapy is limited to psychotropic therapeutic agents.

**Table S4.** *Regression coefficients of the cognitive reappraisal model with brain-PAD, and chronological age, pharmacotherapy, sex assigned at birth, and inhibition.*

*Notes*. ADHD=Attention-deficit/hyperactivity disorder; *b*=unstandardized regression coefficient; Brain-PAD=brain-predicted age difference; CBCL=Child Behavior Checklist; CI=confidence interval; *LL*=lower limit; *p*-values are unadjusted for multiple comparisons; *SE*=standard error; SSRT=Stop Signal Reaction Time (mean estimation); *UL*=upper limit. Non-ADHD pharmacotherapy is limited to psychotropic therapeutic agents.

**Table S5.** *Regression coefficients of the cognitive reappraisal model with brain-PAD, and chronological age, pharmacotherapy, psychological problems, sex assigned at birth, and inhibition.*

*Notes*. ADHD=Attention-deficit/hyperactivity disorder; *b*=unstandardized regression coefficient; Brain-PAD=brain-predicted age difference; CBCL=Child Behavior Checklist; CI=confidence interval; *LL*=lower limit; *p*-values are unadjusted for multiple comparisons; *SE*=standard error; SSRT=Stop Signal Reaction Time (mean estimation); *UL*=upper limit. Non-ADHD pharmacotherapy is limited to psychotropic therapeutic agents.

**Table S6.** *Regression coefficients of the cognitive reappraisal model with brain-PAD, and chronological age, pubertal status, pharmacotherapy, psychological problems, sex assigned at birth, and inhibition.*

*Notes*. ADHD=Attention-deficit/hyperactivity disorder; *b*=unstandardized regression coefficient; Brain-PAD=brain-predicted age difference; CBCL=Child Behavior Checklist; CI=confidence interval; *LL*=lower limit; PDS=Pubertal Development Scale; *p*-values are unadjusted for multiple comparisons; *SE*=standard error; SSRT=Stop Signal Reaction Time (mean estimation); *UL*=upper limit. Non-ADHD pharmacotherapy is limited to psychotropic therapeutic agents.

**Table S7.** *Regression coefficients of the cognitive reappraisal model with brain-PAD, and chronological age, pubertal status, pharmacotherapy, psychological problems, sex assigned at birth, cognitive functioning, and inhibition.*

*Notes*. ^#^=age-corrected standard score; ADHD=Attention-deficit/hyperactivity disorder; *b*=unstandardized regression coefficient; Brain-PAD=brain-predicted age difference; CBCL=Child Behavior Checklist; CI=confidence interval; *LL*=lower limit; PDS=Pubertal Development Scale; *p*-values are unadjusted for multiple comparisons; *SE*=standard error; SSRT=Stop Signal Reaction Time (mean estimation); *UL*=upper limit. Non-ADHD pharmacotherapy is limited to psychotropic therapeutic agents.

**Table S8.** *Regression coefficients of the cognitive reappraisal model with brain-PAD, and chronological age, pubertal status, pharmacotherapy, psychological problems, sex assigned at birth, ethnicity/ race, cognitive functioning, and inhibition.*

*Notes*. ^#^=age-corrected standard score; ADHD=Attention-deficit/hyperactivity disorder; *b*=unstandardized regression coefficient; Brain-PAD=brain-predicted age difference; CBCL=Child Behavior Checklist; CI=confidence interval; *LL*=lower limit; PDS=Pubertal Development Scale; *p*-values are unadjusted for multiple comparisons; *SE*=standard error; SSRT=Stop Signal Reaction Time (mean estimation); *UL*=upper limit. Non-ADHD pharmacotherapy is limited to psychotropic therapeutic agents.

**Table S9.** *Regression coefficients of the expressive suppression model with brain-PAD and chronological age.*

*Notes*. *b*=unstandardized regression coefficient; Brain-PAD=brain-predicted age difference; CI=confidence interval; *LL*=lower limit; *p*-values are unadjusted for multiple comparisons; *SE*=standard error; *UL*=upper limit.

**Table S10.** *Regression coefficients of the expressive suppression model with brain-PAD, and chronological age, pharmacotherapy, and sex assigned at birth.*

*Notes*. ADHD=Attention-deficit/hyperactivity disorder; *b*=unstandardized regression coefficient; Brain-PAD=brain-predicted age difference; CBCL=Child Behavior Checklist; CI=confidence interval; *LL*=lower limit; *p*-values are unadjusted for multiple comparisons; *SE*=standard error; *UL*=upper limit. Non-ADHD pharmacotherapy is limited to psychotropic therapeutic agents.

**Table S11.** *Regression coefficients of the expressive suppression model with brain-PAD, and chronological age, pharmacotherapy, sex assigned at birth, and inhibition.*

*Notes*. ADHD=Attention-deficit/hyperactivity disorder; *b*=unstandardized regression coefficient; Brain-PAD=brain-predicted age difference; CBCL=Child Behavior Checklist; CI=confidence interval; *LL*=lower limit; *p*-values are unadjusted for multiple comparisons; *SE*=standard error; SSRT=Stop Signal Reaction Time (mean estimation); *UL*=upper limit. Non-ADHD pharmacotherapy is limited to psychotropic therapeutic agents.

**Table S12.** *Regression coefficients of the expressive suppression model with brain-PAD, and chronological age, pharmacotherapy, psychological problems, sex assigned at birth, and inhibition.*

*Notes*. ADHD=Attention-deficit/hyperactivity disorder; *b*=unstandardized regression coefficient; Brain-PAD=brain-predicted age difference; CBCL=Child Behavior Checklist; CI=confidence interval; *LL*=lower limit; *p*-values are unadjusted for multiple comparisons; *SE*=standard error; SSRT=Stop Signal Reaction Time (mean estimation); *UL*=upper limit. Non-ADHD pharmacotherapy is limited to psychotropic therapeutic agents.

**Table S13.** *Regression coefficients of the expressive suppression model with brain-PAD, and chronological age, pubertal status, pharmacotherapy, psychological problems, sex assigned at birth, and inhibition.*

*Notes*. ADHD=Attention-deficit/hyperactivity disorder; *b*=unstandardized regression coefficient; Brain-PAD=brain-predicted age difference; CBCL=Child Behavior Checklist; CI=confidence interval; *LL*=lower limit; PDS=Pubertal Development Scale; *p*-values are unadjusted for multiple comparisons; *SE*=standard error; SSRT=Stop Signal Reaction Time (mean estimation); *UL*=upper limit. Non-ADHD pharmacotherapy is limited to psychotropic therapeutic agents.

**Table S14.** *Regression coefficients of the expressive suppression model with brain-PAD, and chronological age, pubertal status, pharmacotherapy, psychological problems, sex assigned at birth, cognitive functioning, and inhibition.*

*Notes*. ^#^=age-corrected standard score; ADHD=Attention-deficit/hyperactivity disorder; *b*=unstandardized regression coefficient; Brain-PAD=brain-predicted age difference; CBCL=Child Behavior Checklist; CI=confidence interval; *LL*=lower limit; PDS=Pubertal Development Scale; *p*-values are unadjusted for multiple comparisons; *SE*=standard error; SSRT=Stop Signal Reaction Time (mean estimation); *UL*=upper limit. Non-ADHD pharmacotherapy is limited to psychotropic therapeutic agents.

**Table S15.** *Regression coefficients of the expressive suppression model with brain-PAD, and chronological age, pubertal status, pharmacotherapy, psychological problems, sex assigned at birth, ethnicity/ race, cognitive functioning, and inhibition.*

*Notes*. ^#^=age-corrected standard score; ADHD=Attention-deficit/hyperactivity disorder; *b*=unstandardized regression coefficient; Brain-PAD=brain-predicted age difference; CBCL=Child Behavior Checklist; CI=confidence interval; *LL*=lower limit; PDS=Pubertal Development Scale; *p*-values are unadjusted for multiple comparisons; *SE*=standard error; SSRT=Stop Signal Reaction Time (mean estimation); *UL*=upper limit. Non-ADHD pharmacotherapy is limited to psychotropic therapeutic agents.

| **Table S1**  *Regression coefficients of the main expressive suppression model without brain-PAD.* | | | | | | | |
| --- | --- | --- | --- | --- | --- | --- | --- |
|  | ERQ Expressive suppression at 3-year follow-up | | | | | | |
|  | *b* | *SE* | 95% CI | | *t* | *p* | |
|  |  |  | *LL* | *UL* |  |  | |
| (Intercept) | 7.175 | 0.964 | 5.284 | 9.066 | 7.439 | | <.001 |
| CBCL ADH Problems (raw scores) | 0.039 | 0.022 | -0.004 | 0.082 | 1.784 | | .075 |
| Pharmacotherapy: ADHD | 0.114 | 0.241 | -0.359 | 0.587 | 0.474 | | .636 |
| Pharmacotherapy: non-ADHD | -0.764 | 0.372 | -1.493 | -0.035 | -2.056 | | .040 |
| Age (in months) | 0.019 | 0.007 | 0.005 | 0.032 | 2.670 | | .008 |
| PDS: early puberty | 0.171 | 0.128 | -0.080 | 0.423 | 1.335 | | .182 |
| PDS: mid puberty | 0.469 | 0.148 | 0.177 | 0.760 | 3.156 | | .002 |
| PDS: late puberty | 1.407 | 0.502 | 0.422 | 2.392 | 2.800 | | .005 |
| Sex assigned at birth: female | -0.115 | 0.116 | -0.343 | 0.112 | -0.992 | | .321 |
| Normalized Euler number | 0.074 | 0.117 | -0.155 | 0.304 | 0.635 | | .526 |
|  | Marginal *R*^2^=.015 | | | | | | |
| *Notes*. *b*=unstandardized regression coefficient; Brain-PAD=brain-predicted age difference; CI=confidence interval; *LL*=lower limit; PDS=Pubertal Development Scale; *p*-values are unadjusted for multiple comparisons; *SE*=standard error; *UL*=upper limit. Non-ADHD pharmacotherapy is limited to psychotropic therapeutic agents. | | | | | | | |

| **Table S2**  *Regression coefficients of the cognitive reappraisal model with brain-PAD and chronological age.* | | | | | | | | |
| --- | --- | --- | --- | --- | --- | --- | --- | --- |
|  | ERQ Cognitive reappraisal at 3-year follow-up | | | | | | | |
|  | *b* | *SE* | 95% CI | | | *t* | *p* | |
|  |  |  | *LL* | | *UL* |  |  | |
| (Intercept) | 9.831 | 0.835 | 8.193 | 11.469 | | 11.770 | | <.001 |
| Brain-PAD | -0.012 | 0.043 | -0.096 | 0.072 | | -0.276 | | .782 |
| Age (in months) | 0.004 | 0.006 | -0.007 | 0.016 | | 0.746 | | .456 |
| Normalized Euler number | 0.042 | 0.100 | -0.154 | 0.238 | | 0.420 | | .675 |
|  | Marginal *R*^2^=.000 | | | | | | | |
| *Notes*. *b*=unstandardized regression coefficient; Brain-PAD=brain-predicted age difference; CI=confidence interval; *LL*=lower limit; *p*-values are unadjusted for multiple comparisons; *SE*=standard error; *UL*=upper limit. | | | | | | | | |

| **Table S3**  *Regression coefficients of the cognitive reappraisal model with brain-PAD, and chronological age, pharmacotherapy, and sex assigned at birth.* | | | | | | | | |
| --- | --- | --- | --- | --- | --- | --- | --- | --- |
|  | ERQ Cognitive reappraisal at 3-year follow-up | | | | | | | |
|  | *b* | *SE* | 95% CI | | | *t* | *p* | |
|  |  |  | *LL* | | *UL* |  |  | |
| (Intercept) | 9.558 | 0.847 | 7.896 | 11.219 | | 11.279 | | <.001 |
| Brain-PAD | -0.007 | 0.043 | -0.091 | 0.077 | | -0.169 | | .866 |
| CBCL ADH Problems (raw scores) | 0.011 | 0.019 | -0.026 | 0.047 | | 0.569 | | .570 |
| Pharmacotherapy: ADHD | -0.331 | 0.207 | -0.737 | 0.076 | | -1.597 | | .110 |
| Pharmacotherapy: non-ADHD | -0.664 | 0.321 | -1.293 | -0.035 | | -2.071 | | .038 |
| Age (in months) | 0.006 | 0.006 | -0.006 | 0.018 | | 0.968 | | .333 |
| Sex assigned at birth: female | 0.078 | 0.089 | -0.096 | 0.253 | | 0.880 | | .379 |
| Normalized Euler number | 0.019 | 0.100 | -0.177 | 0.216 | | 0.194 | | .846 |
|  | Marginal *R*^2^=.004 | | | | | | | |
| *Notes*. ADHD=Attention-deficit/hyperactivity disorder; *b*=unstandardized regression coefficient; Brain-PAD=brain-predicted age difference; CBCL=Child Behavior Checklist; CI=confidence interval; *LL*=lower limit; *p*-values are unadjusted for multiple comparisons; *SE*=standard error; *UL*=upper limit. Non-ADHD pharmacotherapy is limited to psychotropic therapeutic agents. | | | | | | | | |

| **Table S4**  *Regression coefficients of the cognitive reappraisal model with brain-PAD, and chronological age, pharmacotherapy, sex assigned at birth, and inhibition.* | | | | | | | | |
| --- | --- | --- | --- | --- | --- | --- | --- | --- |
|  | ERQ Cognitive reappraisal at 3-year follow-up | | | | | | | |
|  | *b* | *SE* | 95% CI | | | *t* | *p* | |
|  |  |  | *LL* | | *UL* |  |  | |
| (Intercept) | 10.120 | 0.886 | 8.382 | 11.858 | | 11.419 | | <.001 |
| Brain-PAD | -0.008 | 0.043 | -0.092 | 0.076 | | -0.193 | | .847 |
| CBCL ADH Problems (raw scores) | 0.012 | 0.019 | -0.025 | 0.049 | | 0.625 | | .532 |
| Pharmacotherapy: ADHD | -0.346 | 0.207 | -0.753 | 0.061 | | -1.668 | | .096 |
| Pharmacotherapy: non-ADHD | -0.661 | 0.321 | -1.290 | -0.033 | | -2.063 | | .039 |
| Age (in months) | 0.004 | 0.006 | -0.008 | 0.016 | | 0.697 | | .486 |
| Sex assigned at birth: female | 0.080 | 0.089 | -0.094 | 0.255 | | 0.902 | | .367 |
| SSRT | -0.001 | 0.001 | -0.003 | -0.000 | | -2.199 | | .028 |
| Normalized Euler number | 0.014 | 0.100 | -0.182 | 0.211 | | 0.143 | | .886 |
|  | Marginal *R*^2^=.006 | | | | | | | |
| *Notes*. ADHD=Attention-deficit/hyperactivity disorder; *b*=unstandardized regression coefficient; Brain-PAD=brain-predicted age difference; CBCL=Child Behavior Checklist; CI=confidence interval; *LL*=lower limit; *p*-values are unadjusted for multiple comparisons; *SE*=standard error; SSRT=Stop Signal Reaction Time (mean estimation); *UL*=upper limit. Non-ADHD pharmacotherapy is limited to psychotropic therapeutic agents. | | | | | | | | |

| **Table S5**  *Regression coefficients of the cognitive reappraisal model with brain-PAD, and chronological age, pharmacotherapy, psychological problems, sex assigned at birth, and inhibition.* | | | | | | | | |
| --- | --- | --- | --- | --- | --- | --- | --- | --- |
|  | ERQ Cognitive reappraisal at 3-year follow-up | | | | | | | |
|  | *b* | *SE* | 95% CI | | | *t* | *p* | |
|  |  |  | *LL* | | *UL* |  |  | |
| (Intercept) | 10.115 | 0.888 | 8.373 | 11.857 | | 11.385 | | <.001 |
| Brain-PAD | -0.008 | 0.043 | -0.092 | 0.076 | | -0.192 | | .848 |
| CBCL ADH Problems | 0.010 | 0.024 | -0.038 | 0.058 | | 0.423 | | .672 |
| CBCL Total (excluding ADH) Problems | 0.000 | 0.005 | -0.009 | 0.010 | | 0.087 | | .931 |
| Pharmacotherapy: ADHD | -0.345 | 0.208 | -0.753 | 0.062 | | -1.663 | | .096 |
| Pharmacotherapy: non-ADHD | -0.664 | 0.323 | -1.298 | -0.030 | | -2.054 | | .040 |
| Age (in months) | 0.004 | 0.006 | -0.008 | 0.016 | | 0.699 | | .484 |
| Sex assigned at birth: female | 0.080 | 0.089 | -0.095 | 0.254 | | 0.896 | | .370 |
| SSRT | -0.001 | 0.001 | -0.003 | -0.000 | | -2.197 | | .028 |
| Normalized Euler number | 0.014 | 0.100 | -0.183 | 0.211 | | 0.140 | | .889 |
|  | Marginal *R*^2^=.006 | | | | | | | |
| *Notes*. ADHD=Attention-deficit/hyperactivity disorder; *b*=unstandardized regression coefficient; Brain-PAD=brain-predicted age difference; CBCL=Child Behavior Checklist; CI=confidence interval; *LL*=lower limit; *p*-values are unadjusted for multiple comparisons; *SE*=standard error; SSRT=Stop Signal Reaction Time (mean estimation); *UL*=upper limit. Non-ADHD pharmacotherapy is limited to psychotropic therapeutic agents. | | | | | | | | |

| **Table S6**  *Regression coefficients of the cognitive reappraisal model with brain-PAD, and chronological age, pubertal status, pharmacotherapy, psychological problems, sex assigned at birth, and inhibition.* | | | | | | | | |
| --- | --- | --- | --- | --- | --- | --- | --- | --- |
|  | ERQ Cognitive reappraisal at 3-year follow-up | | | | | | | |
|  | *b* | *SE* | 95% CI | | | *t* | *p* | |
|  |  |  | *LL* | | *UL* |  |  | |
| (Intercept) | 10.452 | 0.908 | 8.671 | 12.233 | | 11.506 | | <.001 |
| Brain-PAD | -0.016 | 0.043 | -0.100 | 0.069 | | -0.360 | | .719 |
| CBCL ADH Problems | 0.010 | 0.024 | -0.038 | 0.058 | | 0.416 | | .677 |
| CBCL Total (excluding ADH) Problems | 0.000 | 0.005 | -0.009 | 0.009 | | 0.000 | | 1 |
| Pharmacotherapy: ADHD | -0.340 | 0.208 | -0.747 | 0.067 | | -1.638 | | .102 |
| Pharmacotherapy: non-ADHD | -0.673 | 0.323 | -1.307 | -0.039 | | -2.082 | | .037 |
| Age (in months) | 0.001 | 0.006 | -0.011 | 0.014 | | 0.233 | | .816 |
| PDS: early puberty | 0.028 | 0.110 | -0.188 | 0.244 | | 0.256 | | .798 |
| PDS: mid puberty | 0.233 | 0.126 | -0.014 | 0.480 | | 1.850 | | .064 |
| PDS: late puberty | 0.339 | 0.433 | -0.510 | 1.188 | | 0.782 | | .434 |
| Sex assigned at birth: female | -0.007 | 0.100 | -0.204 | 0.189 | | -0.071 | | .943 |
| SSRT | -0.001 | 0.001 | -0.003 | -0.000 | | -2.225 | | .026 |
| Normalized Euler number | 0.016 | 0.100 | -0.180 | 0.213 | | 0.161 | | .872 |
|  | Marginal *R*^2^=.008 | | | | | | | |
| *Notes*. ADHD=Attention-deficit/hyperactivity disorder; *b*=unstandardized regression coefficient; Brain-PAD=brain-predicted age difference; CBCL=Child Behavior Checklist; CI=confidence interval; *LL*=lower limit; PDS=Pubertal Development Scale; *p*-values are unadjusted for multiple comparisons; *SE*=standard error; SSRT=Stop Signal Reaction Time (mean estimation); *UL*=upper limit. Non-ADHD pharmacotherapy is limited to psychotropic therapeutic agents. | | | | | | | | |

| **Table S7**  *Regression coefficients of the cognitive reappraisal model with brain-PAD, and chronological age, pubertal status, pharmacotherapy, psychological problems, sex assigned at birth, cognitive functioning, and inhibition.* | | | | | | | | |
| --- | --- | --- | --- | --- | --- | --- | --- | --- |
|  | ERQ Cognitive reappraisal at 3-year follow-up | | | | | | | |
|  | *b* | *SE* | 95% CI | | | *t* | *p* | |
|  |  |  | *LL* | | *UL* |  |  | |
| (Intercept) | 10.270 | 0.951 | 8.406 | 12.135 | | 10.802 | | <.001 |
| Brain-PAD | -0.017 | 0.043 | -0.102 | 0.068 | | -0.391 | | .696 |
| CBCL ADH Problems | 0.011 | 0.025 | -0.037 | 0.059 | | 0.455 | | .649 |
| CBCL Total (excluding ADH) Problems | 0.000 | 0.005 | -0.009 | 0.009 | | 0.029 | | .977 |
| Pharmacotherapy: ADHD | -0.333 | 0.208 | -0.741 | 0.074 | | -1.604 | | .109 |
| Pharmacotherapy: non-ADHD | -0.674 | 0.323 | -1.307 | -0.040 | | -2.085 | | .037 |
| Age (in months) | 0.001 | 0.006 | -0.011 | 0.013 | | 0.146 | | .884 |
| PDS: early puberty | 0.032 | 0.110 | -0.184 | 0.248 | | 0.294 | | .769 |
| PDS: mid puberty | 0.243 | 0.127 | -0.005 | 0.492 | | 1.920 | | .055 |
| PDS: late puberty | 0.344 | 0.434 | -0.507 | 1.194 | | 0.792 | | .428 |
| Sex assigned at birth: female | -0.014 | 0.100 | -0.211 | 0.183 | | -0.142 | | .887 |
| Cognition: Crystallized Composite^#^ | -0.001 | 0.003 | -0.006 | 0.004 | | -0.416 | | .678 |
| Cognition: Fluid Composite^#^ | 0.003 | 0.003 | -0.002 | 0.009 | | 1.155 | | .248 |
| SSRT | -0.001 | 0.001 | -0.002 | -0.000 | | -2.081 | | .038 |
| Normalized Euler number | 0.016 | 0.100 | -0.180 | 0.213 | | 0.161 | | .872 |
|  | Marginal *R*^2^=.008 | | | | | | | |
| *Notes*. ^#^=age-corrected standard score; ADHD=Attention-deficit/hyperactivity disorder; *b*=unstandardized regression coefficient; Brain-PAD=brain-predicted age difference; CBCL=Child Behavior Checklist; CI=confidence interval; *LL*=lower limit; PDS=Pubertal Development Scale; *p*-values are unadjusted for multiple comparisons; *SE*=standard error; SSRT=Stop Signal Reaction Time (mean estimation); *UL*=upper limit. Non-ADHD pharmacotherapy is limited to psychotropic therapeutic agents. | | | | | | | | |

| **Table S8**  *Regression coefficients of the cognitive reappraisal model with brain-PAD, and chronological age, pubertal status, pharmacotherapy, psychological problems, sex assigned at birth, ethnicity/ race, cognitive functioning, and inhibition.* | | | | | | | | |
| --- | --- | --- | --- | --- | --- | --- | --- | --- |
|  | ERQ Cognitive reappraisal at 3-year follow-up | | | | | | | |
|  | *b* | *SE* | 95% CI | | | *t* | *p* | |
|  |  |  | *LL* | | *UL* |  |  | |
| (Intercept) | 10.075 | 0.967 | 8.178 | 11.972 | | 10.414 | | <.001 |
| Brain-PAD | -0.035 | 0.044 | -0.121 | 0.050 | | -0.808 | | .419 |
| CBCL ADH Problems | 0.006 | 0.025 | -0.042 | 0.054 | | 0.240 | | .810 |
| CBCL Total (excluding ADH) Problems | 0.001 | 0.005 | -0.008 | 0.011 | | 0.305 | | .761 |
| Pharmacotherapy: ADHD | -0.330 | 0.208 | -0.737 | 0.078 | | -1.587 | | .113 |
| Pharmacotherapy: non-ADHD | -0.684 | 0.323 | -1.318 | -0.050 | | -2.117 | | .034 |
| Age (in months) | 0.001 | 0.006 | -0.011 | 0.013 | | 0.170 | | .865 |
| PDS: early puberty | 0.007 | 0.111 | -0.210 | 0.225 | | 0.064 | | .949 |
| PDS: mid puberty | 0.176 | 0.131 | -0.080 | 0.433 | | 1.348 | | .178 |
| PDS: late puberty | 0.212 | 0.438 | -0.646 | 1.070 | | 0.485 | | .628 |
| Sex assigned at birth: female | -0.007 | 0.101 | -0.204 | 0.191 | | -0.068 | | .945 |
| Cognition: Crystallized Composite^#^ | -0.000 | 0.003 | -0.005 | 0.005 | | -0.087 | | .931 |
| Cognition: Fluid Composite^#^ | 0.004 | 0.003 | -0.002 | 0.010 | | 1.328 | | .184 |
| Race/Ethnicity: Asian | 0.642 | 0.320 | 0.015 | 1.269 | | 2.009 | | .045 |
| Race/Ethnicity: Black | 0.437 | 0.176 | 0.093 | 0.782 | | 2.489 | | .013 |
| Race/Ethnicity: Hispanic | 0.008 | 0.122 | -0.232 | 0.248 | | 0.066 | | .947 |
| Race/Ethnicity: Other | 0.239 | 0.143 | -0.042 | 0.520 | | 1.667 | | .096 |
| SSRT | -0.001 | 0.001 | -0.002 | -0.000 | | -2.029 | | .043 |
| Normalized Euler number | 0.026 | 0.100 | -0.171 | 0.223 | | 0.259 | | .796 |
|  | Marginal *R*^2^=.013 | | | | | | | |
| *Notes*. ^#^=age-corrected standard score; ADHD=Attention-deficit/hyperactivity disorder; *b*=unstandardized regression coefficient; Brain-PAD=brain-predicted age difference; CBCL=Child Behavior Checklist; CI=confidence interval; *LL*=lower limit; PDS=Pubertal Development Scale; *p*-values are unadjusted for multiple comparisons; *SE*=standard error; SSRT=Stop Signal Reaction Time (mean estimation); *UL*=upper limit. Non-ADHD pharmacotherapy is limited to psychotropic therapeutic agents. | | | | | | | | |

| **Table S9**  *Regression coefficients of the expressive suppression model with brain-PAD and chronological age.* | | | | | | | | |
| --- | --- | --- | --- | --- | --- | --- | --- | --- |
|  | ERQ Expressive suppression at 3-year follow-up | | | | | | | |
|  | *b* | *SE* | 95% CI | | | *t* | *p* | |
|  |  |  | *LL* | | *UL* |  |  | |
| (Intercept) | 5.672 | 0.987 | 3.737 | 7.608 | | 5.747 | | **<.001** |
| Brain-PAD | 0.172 | 0.051 | 0.072 | 0.272 | | 3.378 | | **.001** |
| Age (in months) | 0.032 | 0.007 | 0.018 | 0.045 | | 4.483 | | **<.001** |
| Normalized Euler number | 0.049 | 0.118 | -0.182 | 0.280 | | 0.419 | | .675 |
|  | Marginal *R*^2^=.010 | | | | | | | |
| *Notes*. *b*=unstandardized regression coefficient; Brain-PAD=brain-predicted age difference; CI=confidence interval; *LL*=lower limit; *p*-values are unadjusted for multiple comparisons; *SE*=standard error; *UL*=upper limit. | | | | | | | | |

| **Table S10**  *Regression coefficients of the expressive suppression model with brain-PAD, and chronological age, pharmacotherapy, and sex assigned at birth.* | | | | | | | | |
| --- | --- | --- | --- | --- | --- | --- | --- | --- |
|  | ERQ Expressive suppression at 3-year follow-up | | | | | | | |
|  | *b* | *SE* | 95% CI | | | *t* | *p* | |
|  |  |  | *LL* | | *UL* |  |  | |
| (Intercept) | 5.334 | 1.003 | 3.368 | 7.300 | | 5.321 | | **<.001** |
| Brain-PAD | 0.181 | 0.051 | 0.081 | 0.281 | | 3.545 | | **<.001** |
| CBCL ADH Problems | 0.044 | 0.022 | 0.001 | 0.087 | | 2.018 | | **.044** |
| Pharmacotherapy: ADHD | 0.115 | 0.241 | -0.358 | 0.588 | | 0.476 | | .634 |
| Pharmacotherapy: non-ADHD | -0.826 | 0.372 | -1.555 | -0.097 | | -2.222 | | **.026** |
| Age (in months) | 0.033 | 0.007 | 0.019 | 0.047 | | 4.644 | | **<.001** |
| Sex assigned at birth: female | 0.084 | 0.103 | -0.119 | 0.286 | | 0.809 | | .418 |
| Normalized Euler number | 0.033 | 0.118 | -0.200 | 0.265 | | 0.275 | | .784 |
|  | Marginal *R*^2^=.014 | | | | | | | |
| *Notes*. ADHD=Attention-deficit/hyperactivity disorder; *b*=unstandardized regression coefficient; Brain-PAD=brain-predicted age difference; CBCL=Child Behavior Checklist; CI=confidence interval; *LL*=lower limit; *p*-values are unadjusted for multiple comparisons; *SE*=standard error; *UL*=upper limit. Non-ADHD pharmacotherapy is limited to psychotropic therapeutic agents. | | | | | | | | |

| **Table S11**  *Regression coefficients of the expressive suppression model with brain-PAD, and chronological age, pharmacotherapy, sex assigned at birth, and inhibition.* | | | | | | | | |
| --- | --- | --- | --- | --- | --- | --- | --- | --- |
|  | ERQ Expressive suppression at 3-year follow-up | | | | | | | |
|  | *b* | *SE* | 95% CI | | | *t* | *p* | |
|  |  |  | *LL* | | *UL* |  |  | |
| (Intercept) | 5.633 | 1.053 | 3.567 | 7.698 | | 5.347 | | **<.001** |
| Brain-PAD | 0.180 | 0.051 | 0.080 | 0.280 | | 3.523 | | **<.001** |
| CBCL ADH Problems | 0.045 | 0.022 | 0.002 | 0.088 | | 2.041 | | **.041** |
| Pharmacotherapy: ADHD | 0.106 | 0.241 | -0.367 | 0.580 | | 0.441 | | .659 |
| Pharmacotherapy: non-ADHD | -0.823 | 0.372 | -1.552 | -0.094 | | -2.215 | | **.027** |
| Age (in months) | 0.032 | 0.007 | 0.018 | 0.046 | | 4.471 | | **<.001** |
| Sex assigned at birth: female | 0.084 | 0.103 | -0.118 | 0.287 | | 0.816 | | .415 |
| SSRT | -0.001 | 0.001 | -0.002 | 0.001 | | -0.932 | | .351 |
| Normalized Euler number | 0.030 | 0.118 | -0.202 | 0.262 | | 0.256 | | .798 |
|  | Marginal *R*^2^=.014 | | | | | | | |
| *Notes*. ADHD=Attention-deficit/hyperactivity disorder; *b*=unstandardized regression coefficient; Brain-PAD=brain-predicted age difference; CBCL=Child Behavior Checklist; CI=confidence interval; *LL*=lower limit; *p*-values are unadjusted for multiple comparisons; *SE*=standard error; SSRT=Stop Signal Reaction Time (mean estimation); *UL*=upper limit. Non-ADHD pharmacotherapy is limited to psychotropic therapeutic agents. | | | | | | | | |

| **Table S12**  *Regression coefficients of the expressive suppression model with brain-PAD, and chronological age, pharmacotherapy, psychological problems, sex assigned at birth, and inhibition.* | | | | | | | | |
| --- | --- | --- | --- | --- | --- | --- | --- | --- |
|  | ERQ Expressive suppression at 3-year follow-up | | | | | | | |
|  | *b* | *SE* | 95% CI | | | *t* | *p* | |
|  |  |  | *LL* | | *UL* |  |  | |
| (Intercept) | 5.584 | 1.055 | 3.515 | 7.653 | | 5.292 | | **<.001** |
| Brain-PAD | 0.180 | 0.051 | 0.079 | 0.280 | | 3.513 | | **<.001** |
| CBCL ADH Problems | 0.031 | 0.028 | -0.025 | 0.087 | | 1.095 | | .274 |
| CBCL Total (excluding ADH) Problems | 0.004 | 0.005 | -0.007 | 0.015 | | 0.753 | | .451 |
| Pharmacotherapy: ADHD | 0.110 | 0.242 | -0.364 | 0.583 | | 0.454 | | .650 |
| Pharmacotherapy: non-ADHD | -0.860 | 0.375 | -1.595 | -0.125 | | -2.294 | | **.022** |
| Age (in months) | 0.032 | 0.007 | 0.018 | 0.046 | | 4.486 | | **<.001** |
| Sex assigned at birth: female | 0.080 | 0.103 | -0.122 | 0.283 | | 0.777 | | .437 |
| SSRT | -0.001 | 0.001 | -0.002 | 0.001 | | -0.915 | | .360 |
| Normalized Euler number | 0.028 | 0.118 | -0.205 | 0.260 | | 0.233 | | .816 |
|  | Marginal *R*^2^=.014 | | | | | | | |
| *Notes*. ADHD=Attention-deficit/hyperactivity disorder; *b*=unstandardized regression coefficient; Brain-PAD=brain-predicted age difference; CBCL=Child Behavior Checklist; CI=confidence interval; *LL*=lower limit; *p*-values are unadjusted for multiple comparisons; *SE*=standard error; SSRT=Stop Signal Reaction Time (mean estimation); *UL*=upper limit. Non-ADHD pharmacotherapy is limited to psychotropic therapeutic agents. | | | | | | | | |

| **Table S13**  *Regression coefficients of the expressive suppression model with brain-PAD, and chronological age, pubertal status, pharmacotherapy, psychological problems, sex assigned at birth, and inhibition.* | | | | | | | | |
| --- | --- | --- | --- | --- | --- | --- | --- | --- |
|  | ERQ Expressive suppression at 3-year follow-up | | | | | | | |
|  | *b* | *SE* | 95% CI | | | *t* | *p* | |
|  |  |  | *LL* | | *UL* |  |  | |
| (Intercept) | 6.309 | 1.076 | 4.200 | 8.418 | | 5.865 | | **<.001** |
| Brain-PAD | 0.170 | 0.051 | 0.069 | 0.271 | | 3.310 | | **.001** |
| CBCL ADH Problems | 0.032 | 0.028 | -0.024 | 0.088 | | 1.120 | | .263 |
| CBCL Total (excluding ADH) Problems | 0.003 | 0.005 | -0.008 | 0.014 | | 0.538 | | .591 |
| Pharmacotherapy: ADHD | 0.124 | 0.241 | -0.350 | 0.597 | | 0.513 | | .608 |
| Pharmacotherapy: non-ADHD | -0.843 | 0.375 | -1.577 | -0.108 | | -2.249 | | **.025** |
| Age (in months) | 0.025 | 0.007 | 0.011 | 0.040 | | 3.397 | | **.001** |
| PDS: early puberty | 0.177 | 0.128 | -0.075 | 0.429 | | 1.378 | | .168 |
| PDS: mid puberty | 0.462 | 0.149 | 0.170 | 0.753 | | 3.105 | | **.002** |
| PDS: late puberty | 1.227 | 0.504 | 0.238 | 2.217 | | 2.433 | | **.015** |
| Sex assigned at birth: female | -0.094 | 0.116 | -0.322 | 0.133 | | -0.813 | | .416 |
| SSRT | -0.001 | 0.001 | -0.002 | 0.001 | | -0.934 | | .350 |
| Normalized Euler number | 0.016 | 0.118 | -0.216 | 0.248 | | 0.137 | | .891 |
|  | Marginal *R*^2^=.020 | | | | | | | |
| *Notes*. ADHD=Attention-deficit/hyperactivity disorder; *b*=unstandardized regression coefficient; Brain-PAD=brain-predicted age difference; CBCL=Child Behavior Checklist; CI=confidence interval; *LL*=lower limit; PDS=Pubertal Development Scale; *p*-values are unadjusted for multiple comparisons; *SE*=standard error; SSRT=Stop Signal Reaction Time (mean estimation); *UL*=upper limit. Non-ADHD pharmacotherapy is limited to psychotropic therapeutic agents. | | | | | | | | |

| **Table S14**  *Regression coefficients of the expressive suppression model with brain-PAD, and chronological age, pubertal status, pharmacotherapy, psychological problems, sex assigned at birth, cognitive functioning, and inhibition.* | | | | | | | | |
| --- | --- | --- | --- | --- | --- | --- | --- | --- |
|  | ERQ Expressive suppression at 3-year follow-up | | | | | | | |
|  | *b* | *SE* | 95% CI | | | *t* | *p* | |
|  |  |  | *LL* | | *UL* |  |  | |
| (Intercept) | 7.012 | 1.126 | 4.804 | 9.220 | | 6.227 | | **<.001** |
| Brain-PAD | 0.164 | 0.051 | 0.063 | 0.264 | | 3.192 | | **.001** |
| CBCL ADH Problems | 0.024 | 0.029 | -0.032 | 0.080 | | 0.835 | | .404 |
| CBCL Total (excluding ADH) Problems | 0.003 | 0.005 | -0.007 | 0.014 | | 0.637 | | .524 |
| Pharmacotherapy: ADHD | 0.117 | 0.241 | -0.356 | 0.591 | | 0.486 | | .627 |
| Pharmacotherapy: non-ADHD | -0.867 | 0.374 | -1.601 | -0.133 | | -2.315 | | **.021** |
| Age (in months) | 0.026 | 0.007 | 0.012 | 0.041 | | 3.538 | | **<.001** |
| PDS: early puberty | 0.153 | 0.129 | -0.099 | 0.405 | | 1.192 | | .234 |
| PDS: mid puberty | 0.433 | 0.149 | 0.141 | 0.726 | | 2.904 | | **.004** |
| PDS: late puberty | 1.115 | 0.505 | 0.125 | 2.105 | | 2.208 | | **.027** |
| Sex assigned at birth: female | -0.105 | 0.116 | -0.333 | 0.124 | | -0.899 | | .369 |
| Cognition: Crystallized Composite^#^ | -0.009 | 0.003 | -0.015 | -0.004 | | -3.149 | | **.002** |
| Cognition: Fluid Composite^#^ | 0.002 | 0.003 | -0.005 | 0.008 | | 0.495 | | .620 |
| SSRT | -0.001 | 0.001 | -0.002 | 0.001 | | -0.910 | | .363 |
| Normalized Euler number | 0.002 | 0.118 | -0.230 | 0.234 | | 0.014 | | .989 |
|  | Marginal *R*^2^=.024 | | | | | | | |
| *Notes*. ^#^=age-corrected standard score; ADHD=Attention-deficit/hyperactivity disorder; *b*=unstandardized regression coefficient; Brain-PAD=brain-predicted age difference; CBCL=Child Behavior Checklist; CI=confidence interval; *LL*=lower limit; PDS=Pubertal Development Scale; *p*-values are unadjusted for multiple comparisons; *SE*=standard error; SSRT=Stop Signal Reaction Time (mean estimation); *UL*=upper limit. Non-ADHD pharmacotherapy is limited to psychotropic therapeutic agents. | | | | | | | | |

| **Table S15**  *Regression coefficients of the expressive suppression model with brain-PAD, and chronological age, pubertal status, pharmacotherapy, psychological problems, sex assigned at birth, ethnicity/ race, cognitive functioning, and inhibition.* | | | | | | | | |
| --- | --- | --- | --- | --- | --- | --- | --- | --- |
|  | ERQ Expressive suppression at 3-year follow-up | | | | | | | |
|  | *b* | *SE* | 95% CI | | | *t* | *p* | |
|  |  |  | *LL* | | *UL* |  |  | |
| (Intercept) | 5.971 | 1.134 | 3.748 | 8.193 | | 5.267 | | **<.001** |
| Brain-PAD | 0.138 | 0.051 | 0.037 | 0.238 | | 2.687 | | .007 |
| CBCL ADH Problems | 0.019 | 0.028 | -0.037 | 0.075 | | 0.665 | | .506 |
| CBCL Total (excluding ADH) Problems | 0.005 | 0.005 | -0.006 | 0.015 | | 0.850 | | .396 |
| Pharmacotherapy: ADHD | 0.164 | 0.240 | -0.308 | 0.635 | | 0.681 | | .496 |
| Pharmacotherapy: non-ADHD | -0.893 | 0.373 | -1.624 | -0.162 | | -2.396 | | **.017** |
| Age (in months) | 0.027 | 0.007 | 0.013 | 0.042 | | 3.673 | | **<.001** |
| PDS: early puberty | 0.079 | 0.129 | -0.173 | 0.331 | | 0.617 | | .537 |
| PDS: mid puberty | 0.260 | 0.152 | -0.037 | 0.558 | | 1.714 | | .087 |
| PDS: late puberty | 0.817 | 0.506 | -0.174 | 1.809 | | 1.617 | | .106 |
| Sex assigned at birth: female | -0.067 | 0.116 | -0.295 | 0.160 | | -0.580 | | .562 |
| Cognition: Crystallized Composite^#^ | -0.005 | 0.003 | -0.011 | 0.001 | | -1.757 | | .079 |
| Cognition: Fluid Composite^#^ | 0.004 | 0.003 | -0.003 | 0.010 | | 1.037 | | .300 |
| Race/Ethnicity: Asian | 0.491 | 0.378 | -0.250 | 1.232 | | 1.299 | | .194 |
| Race/Ethnicity: Black | 1.164 | 0.210 | 0.753 | 1.575 | | 5.555 | | **<.001** |
| Race/Ethnicity: Hispanic | 0.488 | 0.152 | 0.190 | 0.787 | | 3.210 | | **.001** |
| Race/Ethnicity: Other | 0.404 | 0.168 | 0.075 | 0.732 | | 2.410 | | **.016** |
| SSRT | -0.000 | 0.001 | -0.002 | 0.001 | | -0.606 | | .545 |
| Normalized Euler number | -0.003 | 0.118 | -0.233 | 0.228 | | -0.022 | | .982 |
|  | Marginal *R*^2^=.042 | | | | | | | |
| *Notes*. ^#^=age-corrected standard score; ADHD=Attention-deficit/hyperactivity disorder; *b*=unstandardized regression coefficient; Brain-PAD=brain-predicted age difference; CBCL=Child Behavior Checklist; CI=confidence interval; *LL*=lower limit; PDS=Pubertal Development Scale; *p*-values are unadjusted for multiple comparisons; *SE*=standard error; SSRT=Stop Signal Reaction Time (mean estimation); *UL*=upper limit. Non-ADHD pharmacotherapy is limited to psychotropic therapeutic agents. | | | | | | | | |
